# A New Species of *Boulenophrys* (Megophridae) from Mt. Hengshan, Hunan Province, China, with Re-Description on *B. hengshanensis* †

**DOI:** 10.3390/ani15182745

**Published:** 2025-09-19

**Authors:** Dai-Yong Kuang, Yi-Fu Wei, Yi-Sha Luo, Kang-Wen Pei, Ying-Yue Cao, Meng-Fei Zhang, Tai-Fu Huang, Ling Pu, Sheng-Chao Shi

**Affiliations:** 1 Hubei Engineering Research Center for Protection and Utilization of Special Biological Resources in the Hanjiang River Basin, School of Life Science, Jianghan University, Wuhan 430056, China; 2 Nanyue Arboretum of Hunan Province, Nanyue, Hengyang 421900, China; 3 Administration of Hunan Nanyue Hengshan National Nature Reserve, Nanyue, Hengyang 421900, China; 4 Central South Inventory and Planning Institute of National Forestry and Grassland Administration, Changsha 410014, China; 5 Key Laboratory of Biodiversity Conservation of National Forestry and Grassland Administration, Ecology and Nature Conservation Institute, Chinese Academy of Forestry, Beijing 100091, China

**Keywords:** *Boulenophrys gutu*, *Boulenophrys hengshanensis*, taxonomy, phylogeny, morphology

## Abstract

*Boulenophrys gutu* **sp. nov.** was described from Mt. Hengshan, and *B. hengshanensis* was re-described based on the holotype and newly collected specimens using phylogenetic and morphological evidence. The new species forms an independent clade, and it is diagnosed by a combination of following characters: (1) male SVL 34.4–44.7 mm (*n* = 7), female SVL 36.2–52.8 mm (*n* = 8); (2) dorsal surface of head, body, and limbs relatively smooth; (3) vomerine ridge weak, vomerine teeth absent; (4) narrow lateral fringes on toes; (5) heels moderate long, meeting when thighs are positioned at right angles to body; (6) supratympanic fold behind tympanum thick, distinctly enlarged with thickness near diameter of tympanum; (7) inner metatarsal tubercle small (IMT/SVL 4.4–5.2%); (8) several large dark brown patches along both ventrolateral sides of abdomen; (9) coloration of inner and outer metacarpal tubercle, inner metatarsal tubercle, and tip of digits ivory. Phylogenetic analyses based on 16S rRNA and COI genes revealed that *B. hengshanensis* is sister to *B. wugongensis*. Morphological comparisons showed that *B. hengshanensis* is diagnosed by a combination of following characters: (1) moderate body size, male SVL 34.4–38.0 mm (*n* = 9), female SVL 48.4 mm (*n* = 1); (2) weak vomerine ridge, absence of vomerine teeth; (3) tongue not notched behind; (4) a small horn-like tubercle on upper eyelid; (5) rudimentary webbing between toes; (6) narrow lateral fringes on toes; (7) heels relatively short, not meeting when thighs are positioned at right angles to body; (8) supratympanic fold behind tympanum narrow, not distinctly enlarged; (9) a pair of dark brown broad stripes along ventrolateral sides of abdomen; (10) dense creamy white dots present on lower abdomen, merge with deep brown patches without clear boundary.

## Simple Summary

The genus *Boulenophrys* is a diverse group of Megophryinae found in southeastern Asia and southern China. In 2023, *B. hengshanensis* was described from Mt. Hengshan, Hunan Province, South-central China. However, based on morphological and phylogenetic analyses, we found that the type specimens of recently described species *B. hengshanensis* actually included specimens of two species. Hence, we revised the description and diagnosis of *B. hengshanensis* based on the holotype (HUNL 0706000A), the female paratype (HUNL 1997:6–17), and nine newly collected specimens provided in this study, and described the other species from Mt. Hengshan as *B. gutu* **sp. nov.**

## 1. Introduction

Asian Horned Megophryinae Toads Bonaparte, 1850, is a subfamily found in Tropical Asia from South Asia to China and Southeast Asia with 145 species [1,2,3]. The generic relationship within the subfamily Megophryinae has been revised intensively in recent years [4,5,6,7,8]. In this study, we adopt the ten genera solution recently revised by Lyu et al., which includes *Megophrys* Kuhl and Van Hasselt, 1822, *Xenophrys* Günther, 1864, *Pelobatrachus* Beddard, 1907, *Ophryophryne* Boulenger, 1903, *Atympanophrys* Tian and Hu, 1983, *Brachytarsophrys* Tian and Hu, 1983, *Boulenophrys* Fei, Ye, and Jiang, 2016, Grillitschia Dubois, Ohler, and Pyron, 2021, *Sarawakiphrys* Lyu and Wang, 2023, *Jingophrys* Lyu and Wang, 2023 [2]. With 75 recognized species to date, *Boulenophrys* is the most diverse group among these genera, and still, there are multiple new species being described recently (Table S1) [1,2,3,9,10,11,12].

Mt. Hengshan, also named Nanyue 南岳, is one of the Five Chinese Mountains located in Hengyang City, Hunan Province, China. Two years ago, *Boulenophrys hengshanensis* Qian, Hu, Mo, Gao, Zhang, and Yang, 2023, was described from Mt. Hengshan [13]. Former records of *Boulenophrys brachykolos* (Inger and Romer, 1961) from the mountain were revised as *B. hengshanensis*, leaving the mountain with records of only one species [13].

During our investigations from 2018 to 2025 covering multiple sites on Mt. Hengshan, we collected a series of specimens belonging to *B. hengshanensis* and another species of *Boulenophrys*. Combined morphological data of examined type specimens and newly collected specimens, and phylogenetic analyses based on two mitochondrial genes, we found that the description and diagnosis of *B. hengshanensis* should be revised, and the other species we collected should be assigned to a new species.

## 2. Materials and Methods

### 2.1. Sampling

**Figure 1:**
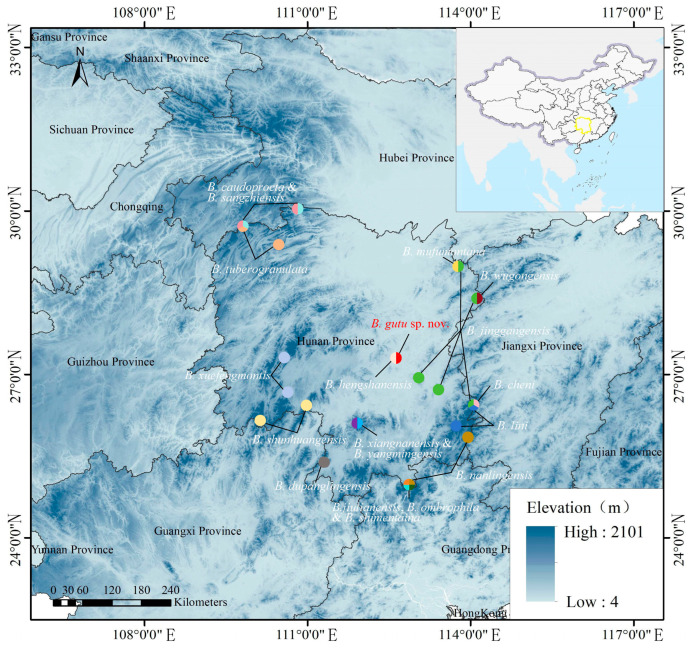
Distribution of recognized *Boulenophrys* species in Hunan Province, China. The red and light pink circle indicates sampling location for this study, Mt. Hengshan, Nanyue District, Hengyang City, Hunan Province, China. Distribution data was based on Lyu et al. [2], Qian et al. [13] and Xiao et al. [11].

Field investigations were conducted in Mt. Hengshan, Nanyue District, Hengyang City, Hunan Province, China (Figure 1) from 2018 to 2025. A total of 24 specimens were collected in this research. They were fixed and preserved in 75% ethanol and subsequently deposited in School of Life Science, Jianghan University (JHUN), Wuhan City, Hubei Province, China, and Chengdu Institute of Biology (CIB), Chinese Academy of Sciences, Chengdu, Sichuan, China. Muscle tissues were sampled from these specimens and stored in 95% ethanol for DNA extraction.

### 2.2. Molecular and Phylogenetic Analyses

Genomic DNA of 15 samples of the candidate new species and nine samples of *Boulenophrys hengshanensis* were extracted by TSINGKE Co., Ltd. (Wuhan, China) (Table S1). Partial 16S ribosomal RNA gene (16S rRNA) and cytochrome c oxidase 1 gene (COI) were sequenced. Primers P7 (5′-CGCCTGTTTACCAAAAACAT-3′) and P8 (5′-CCGGTCTGAACTCAGATCACGT-3′) were used for 16S rRNA following Simon et al. [14]. Primers Chmf4 (5′-TYTCWACWAAYCAYAAAGAYATCGG-3′) and Chmr4 (5′-ACYTCRGGRTGRCCRAARAATCA-3′) were used for COI following Che et al. [15]. Conditions for PCR followed Liu et al. [5].

For phylogenetic analyses, sequences of 77 species were attained from GenBank, including 75 recognized *Boulenophrys* species and two outgroups of genus *Xenophrys* (Table S1). Sequences generated in this research and downloaded were aligned by MEGA11 [16]. Before phylogenetic analysis, ambiguously aligned fragments of one alignment were removed by Gblocks with the following parameter settings: minimum number of sequences for a conserved/flank position (20/20), maximum number of contiguous non-conserved positions (8), minimum length of a block (10), allowed gap positions (with half) [17]. Phylogenetic analyses were conducted using PhyloSuite v1.2.2 based on the concatenated sequences of 16S rRNA and COI fragments [18]. ModelFinder was used to select the best-fit model under BIC criterion [19]. BI phylogeny was inferred using MrBayes 3.2.6 under GTR+I+G+F model (two parallel runs, 10,000,000 generations), in which the initial 25% of sampled data were discarded as burn-in [20]. Maximum likelihood (ML) phylogeny was inferred using IQ-TREE under model GTR+F+I+G4 (16S rRNA) and GTR+F+R4 (COI) for 5000 ultrafast bootstraps [21]. Genetic distances were estimated by MEGA11 using an uncorrected pairwise distance model [16].

### 2.3. Morphological Analysis

Morphological comparison. Ten selected morphological characters separating all 77 currently recognized species of *Boulenophrys* are listed in Table S2 together with their studies. The holotype and a female paratype of *B. hengshanensis* deposited in the Animal Museum of Life Sciences College of Hunan Normal University (HUNL) were examined. The sex of the examined specimens (voucher numbers were included in Table S1) was determined by examination of vocal sac openings or gonads.

Morphometric analysis. Morphological terminologies and methods mostly followed Mahony et al. [22] and Fei and Ye [22,23]. Measurements were taken with a vernier caliper to the nearest 0.1 mm. A total of 27 characters were measured for adult specimens:

**SVL** snout-vent length (from the tip of the snout to the posterior edge of the vent);

**HL** head length (from the rear of the mandible to the tip of the snout);

**HW** head width (distance between the posterior angles of jaw);

**SL** snout length (from tip of snout to anterior border of the orbit);

**SND** nostril–snout distance (from nostril to tip of the snout);

**END** EN eye–nostril distance (from front of eye to the center of nostril);

**IND** internarial distance (the shortest distance between two nostrils);

**IOD** interorbital distance (the shortest distance between inner edge of upper eyelids)

**EL** eye length (the horizontal distance between the anterior corner and posterior corner of orbit);

**UEW** maximum upper eyelid width;

**IFE** internal front of eyes (the shortest distance between the anterior borders of the left and right orbits);

**IBE** internal back of eyes (the shortest distance between the posterior borders of the left and right orbits);

**TYD** largest horizontal tympanum diameter;

**TYE** tympanum–eye distance (distance from the anterior border of the tympanum to the posterior orbital border);

**FAL** forearm length (distance from elbow to wrist);

**HAL** hand length (distance from wrist to tip of third digit);

**FIL** finger I length (from base of finger I to its tip);

**FIIL** finger II length (from base of finger II to its tip);

**FIIIL** finger III length (from base of finger III to its tip);

**FIVL** finger IV length (from base of finger IV to its tip);

**FIIIW** finger III width (largest width of tip of finger III);

**HLL** hindlimb length (from center of vent to tip of toe IV);

**FML** femoral length (from center of vent to outer edge of knee when leg was folded);

**TIL** tibia length (from outer edge of knee to tibio-tarsal joint when leg was folded);

**TFOL** tarsal–foot length (distance from tibio-tarsal joint to tip of fourth toe);

**FOL** foot length (from proximal edge of inner metatarsal tubercle to tip of fourth toe);

**IMT** length of the inner metatarsal tubercle.

The significance of differences in morphometric characters between the two species from Mt. Hengshan were detected using one-way analysis of variance (ANOVA). The significance level was set at 0.05. Principal component analyses (PCA) were performed using R version 4.5.1 (Copyright (C) 2025 The R Foundation for Statistical Computing, Vienna, Austria) based on morphometric characters. To reduce the impact of allometry, the ratio of each character to SVL was calculated (except SVL itself). Then they were log-transformed for subsequent analyses [24].

## 3. Results

### 3.1. Phylogenetic Analyses

**Figure 2:**
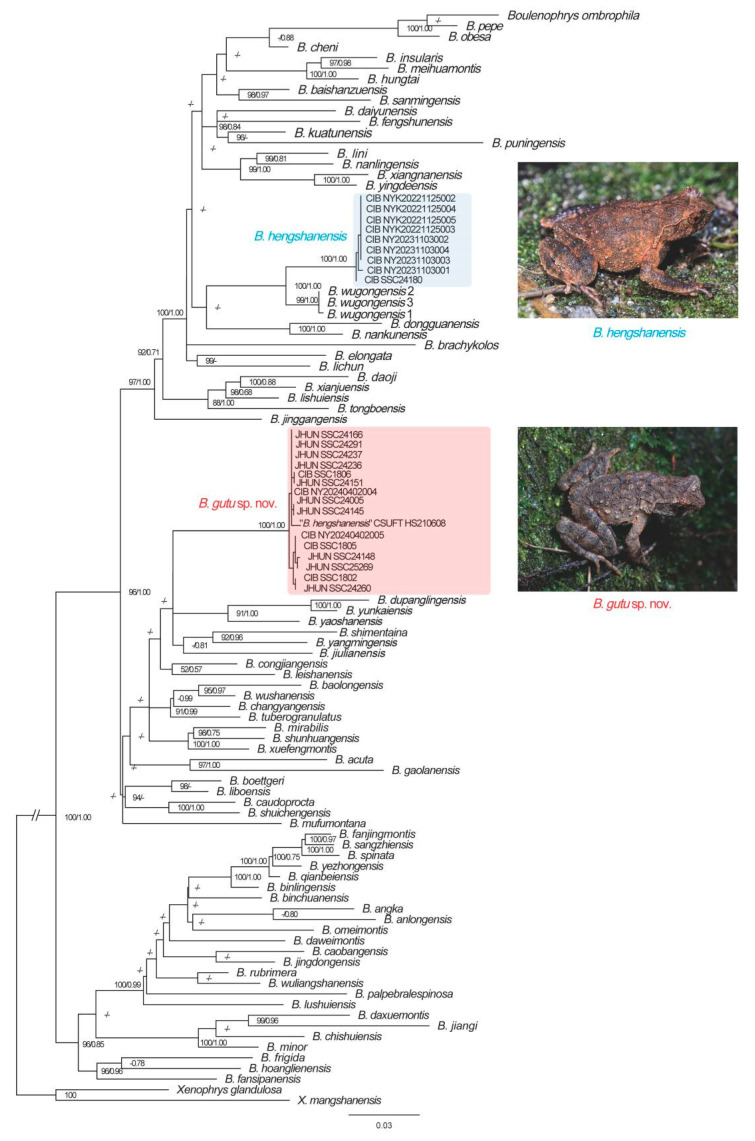
Phylogenetic relationships of the genus *Boulenophrys* based on partial 16S rRNA and COI genes by IQ-TREE. Ultrafast bootstrap approximation (UFB) and Bayesian posterior probabilities (BPP) were denoted beside each node (those lower than 95/0.7 were denoted as “-”).

Analyses were based on sequences with a length of 1243 base pairs (bp), which were concatenated by 571 bp of 16S rRNA sequences and 672 bp of COI sequences. Results of phylogenetic analyses by IQ and BI methods are essentially similar (Figure 2). Fifteen newly collected specimens (e.g., CIB NY20240402004) phylogenetically clustered with “*B. hengshanensis*” (specimens collected in 2021) of Qian et al. [13] and formed an independent clade. Nine specimens (e.g., CIB NYK20221125002) clustered into a clade sister to *B. wugongensis*. The smallest genetic distance between “*B. hengshanensis*” clade and other species based on 16S rRNA gene and COI gene are 1.5% (*B. shunhuangensis*, *B. yunkaiensis*) and 8.5% (*B. congjiangensis)*, respectively, both are among inter-specific level compared with other known species pairs (Tables S3 and S4).

### 3.2. Morphological Analyses

**Figure 3:**
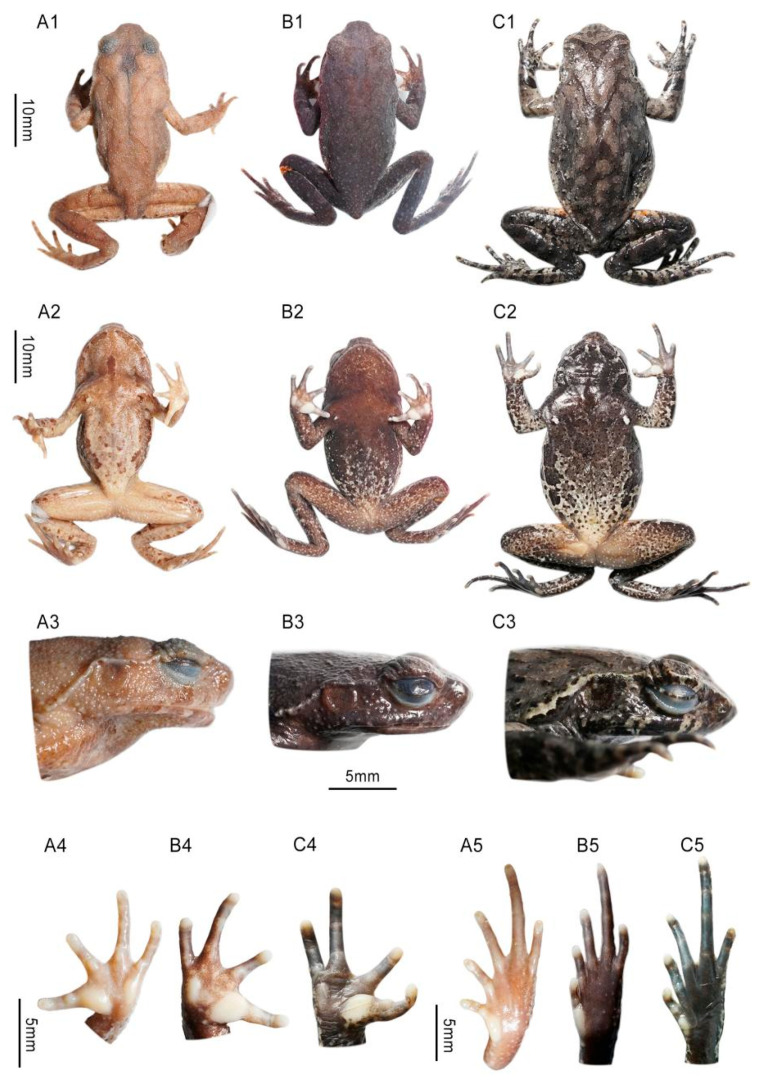
Morphological comparison between adult males of *Boulenophrys hengshanensis* and *B. gutu* sp. nov. (**A**) holotype of *B. hengshanensis* HUNL 0706000A; (**B**) topotype of *B. hengshanensis* CIB NYK20221125003; (**C**) holotype of *B. gutu* sp. nov. CIB NY20240402004; (**1**) dorsal body; (**2**) ventral body; (**3**) lateral head; (**4**) ventral hand; (**5**) ventral feet.

Morphological comparisons between the two species from Mt. Hengshan and all recognized species of *Boulenophrys* suggested that they differ from all recognized species (Table S2). For the two species collected in this study, we found that the morphology of the holotype (HUNL 0706000A) of *B. hengshanensis* do not match the morphology of the 15 specimens in “*B. hengshanensis*” clade in relatively hindlimb length, supratympanic fold, coloration pattern, etc., (e.g., CIB NY20240402004). However, the morphology of nine specimens in the sister clade of *B. wugongensis* (e.g., CIB NYK20221125003) is consistent with the morphology of the holotype of *B. hengshanensis* (Figure 3; see detailed description and comparison in following part). Thus, we identified that the nine specimens in the sister clade of *B. wugongensis* as *B. hengshanensis* according to Article 73 of the code and the 15 specimens in “*B. hengshanensis*” clade represent a candidate new species.

The results of the ANOVA tests revealed significant differences (*p* < 0.05; Table 1) in several morphometric characteristics between males of the candidate new species clade and *B. hengshanensis*. The ratio of END, EL, IFE, TYE, TIL, and IMT to SVL are significantly different among the two species. Detailed measurements are listed in Table S5.

**Figure 4:**
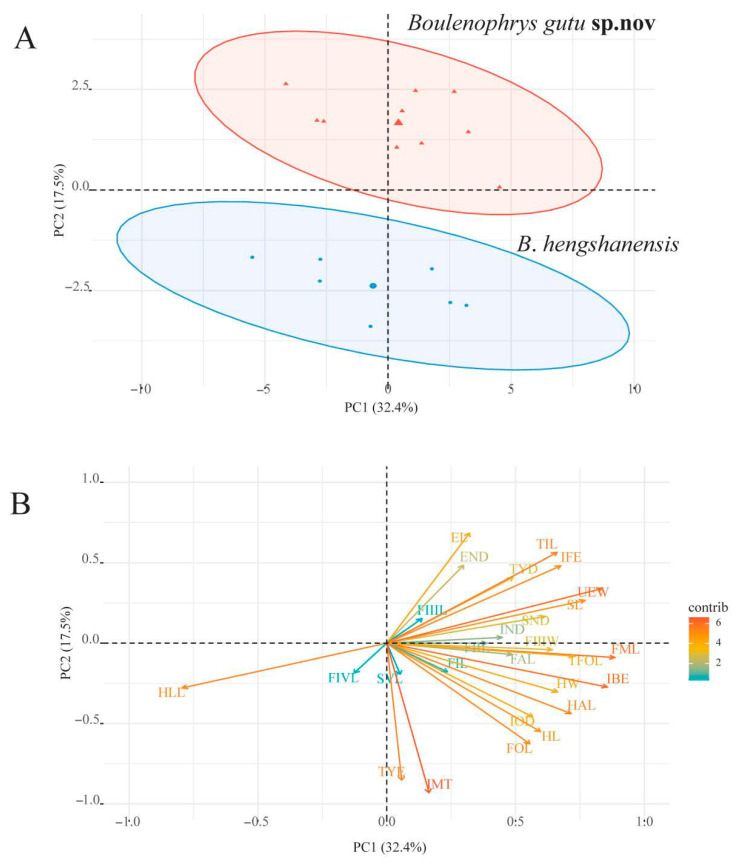
Plots (**A**) and loading diagram (**B**) of the first principal component (PC1) versus the second (PC2) for adult males of *Boulenophrys hengshanensis* and *B. gutu* sp. nov.

In the PCA for males of examined specimens of *Boulenophrys* from Mt. Hengshan in this research, the first two principal components accounted for 49.9% of the total variance. Loadings for PC1, which explained 32.4% of the total variance, were most heavily weighted on EL, TIL, END, IFE, and TYD. Loadings for PC2 explained 21.4% of the total variance and were most heavily weighted on FML and IBE. Differentiation between the two species along the PC1 axis is obvious (Figure 4).

Hence, based on phylogenetical and morphological analyses, we conclude that these 15 specimens in “*B. hengshanensis*” clade represent a new species, and it is described herein.

### 3.3. Taxonomic Account

*Boulenophrys hengshanensis* Qian, Hu, Mo, Gao, Zhang, and Yang, 2023.

**Figure 5:**
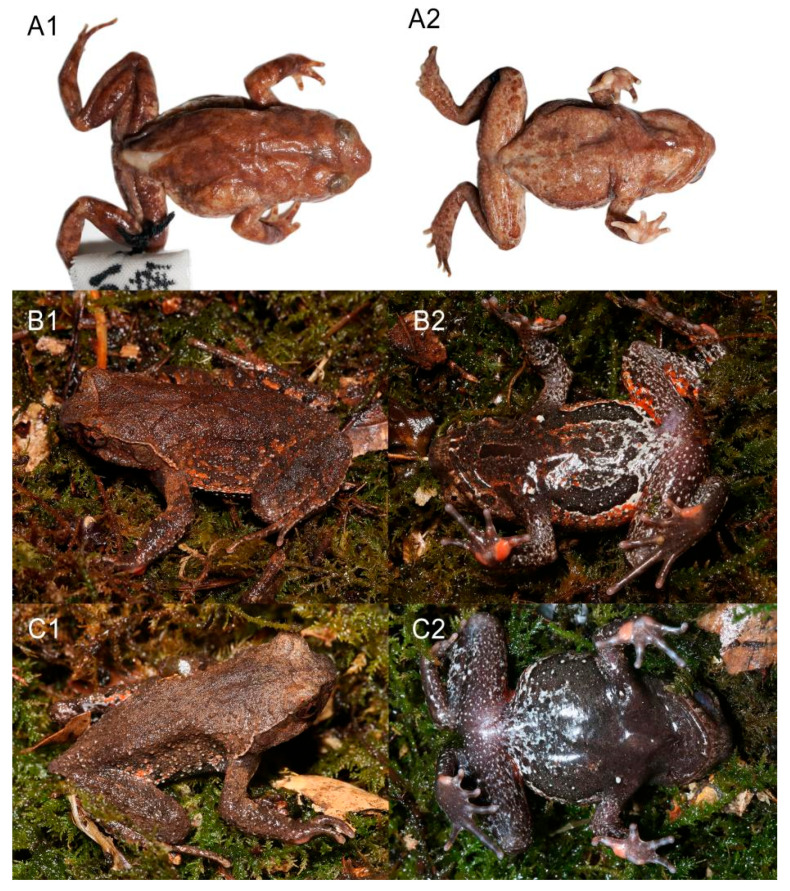
Paratype and topotypes of *Boulenophrys hengshanensis*. (**A**) female paratype HUNL 1997:6–17; (**B**) male topotype CIB NYK20221125005; (**C**) male topotype CIB NY20221125003; (**1**) dorsal or dorsolateral view of body; (**2**) ventral view of body.

Figure 3 and Figure 5.

**Chresonymy.** *Megophrys* brachykolos—Mo et al. [25], Shen et al. [26], Gao et al. [27].

**Holotype.** HUNL 070600A, adult male, collected by Youhui Shen from Mt. Hengshan, Nanyue District, Hengyang City, Hunan Province, China, in June 2007.

**Paratype.** HUNL 1997:6–17, adult female, pregnant, collected by Youhui Shen from Laotayi, Mt. Hengshan, Nanyue District, Hengyang City, Hunan Province, China (near 112.68308° E, 27.27175° N, ca. 980 m a.s.l.), according to the original collection label.

**Remarks.** The description of *B. hengshanensis* was based on two historical specimens preserved in formalin (HUNL 070600A and HUNL 1997:6–17) in Life Science College, Hunan Normal University and sixteen adult males (CSUFT HS210602–603, 605–610, 612–616, and 618–620) and four adult females (CSUFT HS210604, 611, 617, and 621) preserved in 75% alcohol, collected from Yanshou Village, Nanyue District, on 4 June 2021 (according to the corrected coordinate 112.714° E, 27.277° N, ca. 450 m a.s.l., which was incorrectly recorded as 112.714° N, 27.277° E) [13]. The holotype is actually an adult male instead of female as the original description stated with the presence of vocal sac openings [13]. A total of 15 newly collected specimens (e.g., CIB NY20240402004; Table S1) were phylogenetically clustered with the “*B. hengshanensis*” clade (specimens collected in 2021) from Qian et al. [13]. However, the morphology of these specimens do not match with those of unsequenced male holotype HUNL 070600A and the female paratype HUNL 1997:6–17 of *B. hengshanensis* in heels meeting when the thighs are positioned at right angles to then body (vs. not); supratympanic fold behind tympanum thick, distinctly enlarged with thickness near diameter of tympanum (vs. narrow, not distinctly enlarged); several large dark brown patches along both ventrolateral sides of abdomen (vs. dark brown broad stripe); etc., (Figure 4). On the other hand, the nine specimens (e.g., CIB NYK20221125003, Table 1) sister to *B. wugongensis* are morphologically consistent with the male holotype HUNL 070600A and the female paratype HUNL 1997:6–17 of *B. hengshanensis* in characters mentioned above. These specimens were collected at or near the collection site of female paratype HUNL 1997:6–17. Thus, we suggest identifying these specimens as *B. hengshanensis*, while the fifteen specimens (e.g., CIB NYK20221125003) collected in this study and those collected in 2021 by Qian et al. [13] should represent the new species.

**Diagnosis.** *Boulenophrys hengshanensis* differs from its congeners by (1) moderate body size, male SVL 34.4–38.0 mm (*n* = 9), female SVL 48.4 mm (*n* = 1); (2) weak vomerine ridge, an absence of vomerine teeth; (3) tongue not notched behind; (4) a small horn-like tubercle on the upper eyelid; (5) rudimentary webbing between the toes; (6) narrow lateral fringes on the toes; (7) heels relatively short, not meeting when thighs are positioned at right angles to the body; (8) supratympanic fold behind tympanum narrow, not distinctly enlarged; (9) a pair of dark brown broad stripes along ventrolateral sides of the abdomen; (10) dense creamy white dots present on lower abdomen, merge with deep brown patches without clear boundary.

**Description of Holotype.** Measurements in mm. Body size moderate (SVL 37.3), stout; head moderate, wider than long (HW 13.4, HL 12.0, IFE 6.1, IBE 10.9); snout truncated in dorsal view, slight protruding beyond lower jaw in profile (SL 4.4), rostral appendage absent; canthus rostralis distinct; loreal region slightly concave; nostril nearly oval, closer to eye than snout (SND 2.2, END 1.7), distance between nostrils slightly larger than inter orbit distance and wider than upper eyelid (IND 4.9, IOD 3.8, UEW 3.0); tympanum diameter larger than half of eye diameter (EL 4.9, TYD 2.9), and equal to tympanum-eye distance (TYE 1.6); tympanum near rounded, slightly raised above surrounding, upper edge conceal with supratympanic ridge; interorbital space flat; pineal ocellus absent; vomerine ridges weak, while vomerine teeth absent; maxillary teeth present; tongue not notched behind, medial lingual process absent; opening of vocal sac slit like, small, positioned at corner of mouth.

Forelimbs. Forearm moderately long, slightly wider than upper arm, but shorter than hand (FAL 8.1, HAL 9.6); fingers slender, webbing and lateral fringes absent; supernumerary tubercles below the base of all finger small but clear (Figure 3(A4,B4)); subarticular tubercles indistinct, base of first finger distinctly larger than the second; inner and outer metacarpal tubercles slightly raised above hand, independent with each other; finger length formula I < II < IV < III (FIL 2.2, FIIL 2.3, FIIIL 4.0, FIVL 3.3); tip of fingers rounded, not expand to small pad (FIIIW 0.8).

Hindlimbs. Hindlimbs moderate long (HLL 48.3); tibio-tarsal articulation reaches region between eye and tympanum when leg stretched forward along body; heels not meeting when thighs are positioned at right angles to body; thigh longer than tibia and equal to foot (FML 16.4, TIL 15.8, TFOL 22.6, FOL 15.8); toes long, length formula I < II < V < III < IV, rudimentary webbed, lateral fringes on toes narrow, tips rounded and not expanded; indistinct supernumerary tubercles present on base of toe I, II and III; subarticular tubercles absent; outer metatarsal tubercle absent, inner metatarsal tubercle oval, distinct (IMT 3.0).

Skin. Dorsal surface of head, body, and limbs rough, dense granules and sparse small tubercles; several granules present on lateral head below supratympanic ridges and around tympanum; a small tubercle present on outer edge of upper eyelid; supratympanic ridges distinct and narrow, curve above tympanum to shoulder, posterior part behind tympanum not distinctly enlarged; dorsolateral skin fold on body disconnected; longitudinal narrow skin ridge present on dorsum in “>-<” shape, but absent on head; a relatively larger tubercle present on brown transverse bands on dorsal thigh and tibia; several larger tubercles present on flanks with width similar to finger tips. Most ventral surface of body and limbs smooth; skin on throat and abdomen covered with flat granules; pectoral gland flat, small, width similar to a fingertip; femoral gland small, closer to outer edge of knee than cloaca; small granules present around cloaca.

Coloration in preservative. Dorsal body uniformly light brown; dorsal arm and hindlimb basically light brown with transverse brown narrow bands, two on arm, three on each thigh and tibia. Ventral body lighter than dorsal, skin on lower jaw and upper chest light brown, several brown patches present on mandible, a short longitudinal brown bar present on throat; a pair of dark brown broad stripe present along ventrolateral sides of abdomen from near armpit to groin; center of abdomen marbled with sparse small brown patches; ventral surface of limbs ivory tanned with brown pigments, front surface of thigh with several round patches; ventral hand pale, ventral feet light brown.

**Coloration in life.** Description based on nine male specimens listed in Table 1. Dorsal body uniformly brown or brown with irregular small orange patches on flanks (Figure 5); deep brown longitudinal “>-<” pattern on dorsum indistinct or absent; iris copper; small tubercle on the upper eyelid orange; ventral surface of the head brown with several brown patches present on the mandible, a short longitudinal brown bar present on the throat; the ventral head of some individual uniformly deep brown; a pair of deep brown broad stripe present along the ventrolateral sides of abdomen from near armpit to groin, merging with deep brown dark patches on the center of the abdomen on some individuals; dense creamy white dots present on lower abdomen, surrounding deep brown broad stripe, but merging with deep brown patches without a clear boundary; sparse creamy white dots scattered on ventral limbs; skin on groin region orange; ventral hand and feet mostly deep brown; inner metacarpal tubercle, outer metacarpal tubercle, and inner metatarsal tubercle orange; tips of fingers and toes light orange; pectoral and femoral gland creamy white.

**Sexual secondary characteristics.** Adult female with larger body size (Table 1 and Table S5, Figure 5); nuptial pad with dense tiny spines present on dorsal surface of inner two fingers of breeding males; single internal subgular vocal sac present in males; forearm slightly enlarged in adult males.

**Comparisons.** Phylogenetically, *Boulenophrys hengshanensis* is sister to *B. wugongensis*, it differs from the latter in larger body size (SVL 34.4–38.0 mm in nine adult males, 48.4 mm in one adult female vs. 31.0–34.1 mm in four adult males, 38.5–42.8 mm in nine adult females); dense creamy white dots present on the lower abdomen, merging with deep brown patches without clear boundary (vs. clear and large creamy white nebulous patches); narrow lateral fringes on toes present (vs. lateral fringes on toes absent). Comparisons with other recognized species were presented in Table S2.

**Ecological notes.** *B. hengshanensis* inhabits moist montane forest and bush near the top of Mt. Hengshan, only found near streams from elevation ca. 700 m to ca. 1100 m. Advertisement calls were heard from October to April. *Quasipaa boulengeri* (Günther, 1889), *Amolops sinensis* Lyu, Wang and Wang, 2019, and the other species of *Boulenophrys* from Mt. Hengshan were found to be sympatric with *B. hengshanensis.*

*Boulenophrys gutu* sp. nov. Kuang, Wei, and Shi

**Figure 6:**
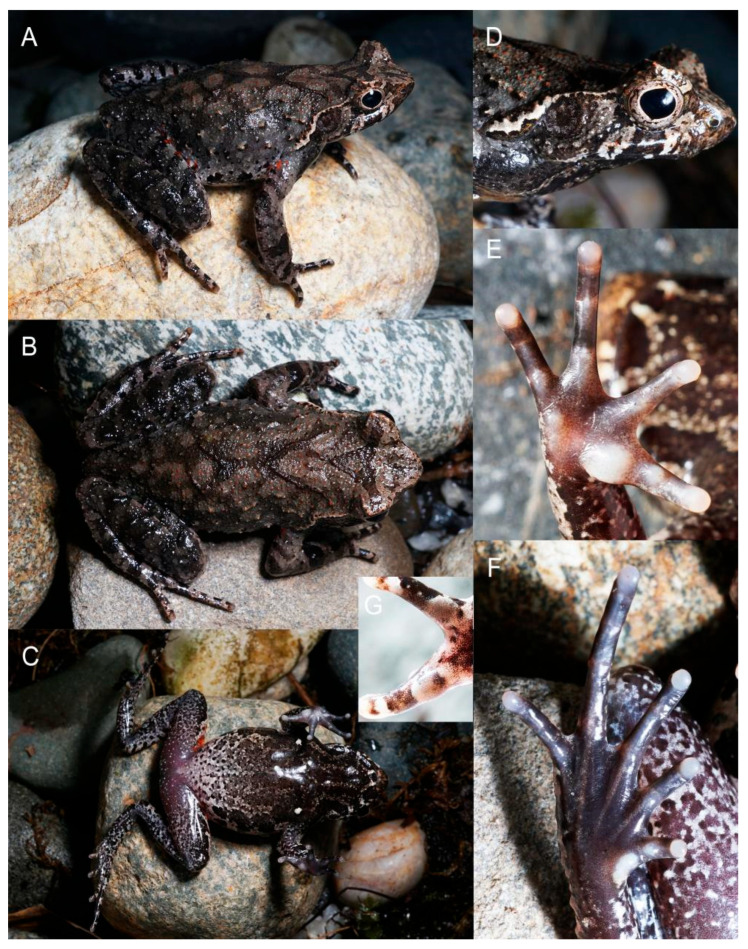
Holotype of *Boulenophrys gutu* sp. nov. CIB NY20240402004 in life. (**A**) dorsolateral body; (**B**) dorsal body; (**C**) ventral body; (**D**) lateral head; (**E**) ventral hand; (**F**) ventral feet; (**G**) nuptial pads on inner two fingers.

**Figure 7:**
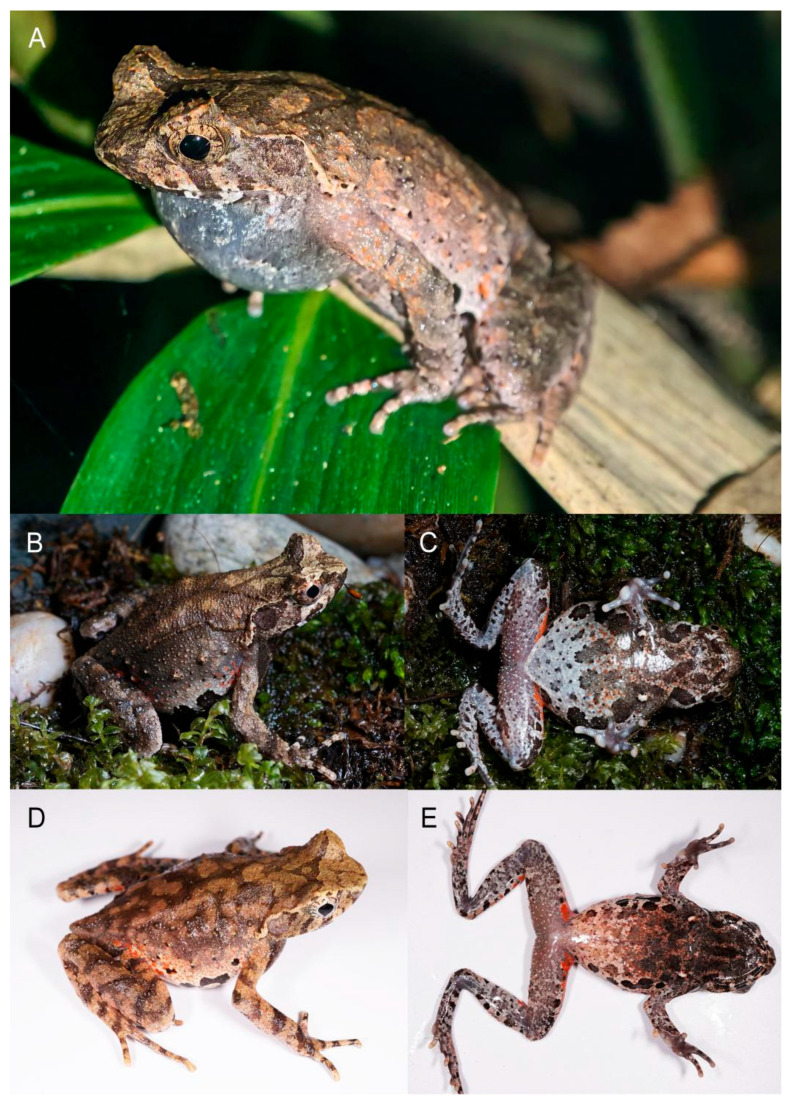
Paratypes of *Boulenophrys gutu* **sp. nov.** in life. (**A**) male paratype JHUN SSC24148 calling on a bamboo leaf; (**B**,**C**) dorsolateral and ventral view of female paratype CIB NY20240402005; (**D**,**E**) dorsolateral and ventral view of female paratype CIB SSC1804.

Figure 3, Figure 6 and Figure 7.

https://zoobank.org/References/8C61C98E-20CA-46ED-8F57-803BA9AB47FD;

https://zoobank.org/NomenclaturalActs/BBB25DD1-125C-4032-9D0C-72EBA75C678A.

**Chresonymy.** *Boulenophrys* hengshanensis—Qian et al. [13].

**Holotype.** CIB NY20240402004, adult male, collected by Shengchao Shi and Daiyong Kuang from Maguxianjing, Yanshou Village, Nanyue Town, Nayue District, Hengyang City, Hunan Province, China (南岳镇延寿村麻姑仙境112.6993° E, 27.2734° N, ca. 670 m a.s.l.) on 2 April 2024.

**Paratype.** A total of six male paratypes: four adult males (JHUN SSC24236–237, JHUN SSC24260, JHUN SSC24291) collected from Yanshou village by Dai-Yong Kuang in April 2023 and May 2024; two adult males (JHUN SSC24148 and JHUN SSC24151) were collected by Sheng-Chao Shi near Cangjing Temple, Longchi Village, Shouyue Town, Nayue District, Hengyang City, Hunan Province, China (112.6784° E, 27.2732° N, ca.1045 m a.s.l.) on 26 July 2024. A total of eight adult female paratypes; CIB NY20240402005 was collected along with the holotype; three adult females (CIB SSC1806, CIB SSC1802, and CIB SSC1805) were collected from the site of holotype by Sheng-Chao Shi on 27 April 2018; JHUN SSC24005 was collected from Xinglong Village, Nanyue District (112.6808° E, 27.2418° N, ca. 500 m a.s.l.) by Sheng-Chao Shi on 19 July 2024; JHUN SSC24166 was collected from the site of holotype by Sheng-Chao Shi on 29 July 2024; JHUN SSC24145 was collected near Cangjing Temple by Sheng-Chao Shi on 26 July 2024; JHUN SSC25269 was collected near, Tanfo Temple, Shouyue Town, Nayue District (112.6645° E, 27.2377° N, ca. 919 m a.s.l.) by Sheng-Chao Shi on 4 May 2025.

**Diagnosis.** *Boulenophrys gutu* sp. nov. is distinguished from congeners by a combination of following characters: (1) male SVL 34.4–44.7 mm (*n* = 7), female SVL 36.2–52.8 mm (*n* = 8); (2) dorsal surface of the head, body, and limbs relatively smooth; (3) vomerine ridge weak, vomerine teeth absent; (4) narrow lateral fringes on toes; (5) heels moderate long, meeting when thighs are positioned at right angles to body; (6) supratympanic fold behind tympanum thick, distinctly enlarged with thickness near diameter of tympanum; (7) inner metatarsal tubercle small (IMT/SVL 4.4–5.2%); (8) several large dark brown patches along both ventrolateral sides of abdomen; (9) coloration of inner and outer metacarpal tubercle, inner metatarsal tubercle, and tip of digits ivory.

**Description of Holotype.** Measurements in mm. Body size moderate (SVL 44.7, Figure 6), stout; head moderate, wider than long (HW 15.4, HL 13.1, IFE 8.3, IBE 12.1); snout near rounded in dorsal view, protruding beyond the lower jaw in profile (SL 5.3), rostral appendage absent; canthus rostralis distinct; loreal region slightly concave; nostril nearly oval, slightly closer to the eye than the snout (SND 2.6, END 2.5), distance between the nostrils smaller than inter orbit distance and wider than upper eyelid (IND 4.0, IOD 4.3, UEW 3.9); tympanum diameter larger than half of eye diameter (EL 5.3, TYD 3.0), and larger than the tympanum-eye distance (TYE 2.8); tympanum near rounded, slightly raised, upper edge conceal with supratympanic ridge; interorbital space flat; pineal ocellus absent; vomerine ridges weak, vomerine teeth absent; maxillary teeth present; tongue not notched behind, medial lingual process absent; opening of vocal sac slit like, small, positioned at corner of mouth.

Forelimbs. Forearm moderately long, slightly enlarged than upper arm, shorter than hand (FAL 9.7, HAL 10.4); fingers slender, without webbing and lateral fringes; supernumerary tubercles present below the base of all finger; subarticular tubercles indistinct, base of first finger distinctly larger than the second; inner and outer metacarpal tubercles slightly raised above hand, independent with each other; finger length formula II<I<IV<III (FIL 2.9, FIIL 2.6, FIIIL 5.4, FIVL 3.5); tip of fingers rounded, not expand to small pad (FIIIW 1.0).

Hindlimbs. Hindlimbs slender (HLL 61.5); tibio-tarsal articulation reaches region between the eye and tympanum when the leg stretched forward along the body; heels meeting when thighs are positioned at right angles to the body; the thigh longer than the tibia and equal to the foot (FML 18.7, TIL 19.4, TFOL 27, FOL 16.6); toes long, length formula I < II < V < III < IV, rudimentary webbed, with indistinct narrow lateral fringes, tips rounded and not expanded; indistinct supernumerary tubercles present on base of toe I, II and III; subarticular tubercles absent; outer metatarsal tubercle absent, inner metatarsal tubercle oval, small (IMT 1.8).

Skin. Dorsal skin of the head, body, and limbs relatively smooth, scattered with sparse granules; several granules present on lateral head below supratympanic ridges and around tympanum; a small horn-like tubercle present on outer edge of upper eyelid; supratympanic ridges distinct, curve above tympanum to shoulder, posterior part behind tympanum distinctly enlarged with thickness near diameter of tympanum; dorsolateral skin fold on body absent; a longitudinal “> <” shaped narrow skin ridge present on dorsum; a triangle skin weak ridge present on head between eyes absent; a relatively larger tubercle present on brown transverse bands on dorsal thigh and tibia; several large tubercles present on flanks with width similar to finger tips. The skin on ventral surface of body and limbs smooth; the pectoral gland flat, distinct, wider than fingertip; femoral gland small, closer to outer edge of knee than cloaca; small granules present around cloaca.

Coloration in life (Figure 6). The dorsal body basically grayish brown; granules on the dorsal body and the head orange; deep brown inverted triangular pattern present on the head and longitudinal “> <” pattern present on dorsum; iris copper; small horn-like tubercle on upper eyelid orange; supratympanic ridges white; five or six deep brown transverse bands present on dorsal thigh, tibia; ventral surface of head deep brown with several indistinct stripes; four pair of large deep brown patches present along ventrolateral sides of abdomen from near the armpit to the groin; other area of abdomen marbled with cream white, light brown and deep brown gravel mosaics, coloration generally whiter on lower abdomen; skin on groin region orange; ventral limbs also marbled with cream white, light brown and deep brown gravel mosaics; the skin around cloaca purplish; ventral hand mostly ivory while ventral feet mostly brown; inner metacarpal tubercle, outer metacarpal tubercle, inner metatarsal tubercle, and tip of fingers and toes ivory; pectoral and femoral gland creamy white.

Coloration in preservative. The skin and granules with orange color turned into ivory. The dorsal head, body, and limbs grayish brown; triangular pattern on head, longitudinal “> <” pattern on dorsum, and cross bands on limbs deep brown. Ventral body lighter than dorsal.

**Sexual secondary characteristics.** Adult female with larger body size (Supplementary Table S1, Figure 6); nuptial pad with dense tiny spines present on inner two fingers of breeding males; single internal subgular vocal sac present in males (Figure 7); forearm slightly enlarged in adult males.

**Etymology.** The specific epithet gutu (顾莵 in Chinese) refers to the “toad” on the moon in ancient Chinese poetry *Tian Wen* (天问) by Qu Yuan (屈原, 340–278 BC) of Chu State (楚国) in the Warring States Period. The poetry documented many questions about nature, including why there is “toad” on the moon (厥利维何，而顾菟在腹). This species is named to commemorate the shared spirit of exploration of humankind. We suggest $\overset{ɡù}{顾}$$\overset{tù}{莵}$$\overset{jiǎo}{角}$$\overset{chán}{蟾}$ as the Chinese name.

**Comparisons.** *Boulenophrys gutu* **sp. nov.** was confused with partially sympatric species *B. hengshanensis*, but differs in multiple characters: heels meeting when thighs are positioned at right angles to body (vs. not); dorsal surface of head, body and limbs relatively smooth (vs. rough); supratympanic fold behind tympanum thick, distinctly enlarged with thickness near diameter of tympanum (vs. narrow, not distinctly enlarged); inner metatarsal tubercle small (IMT/SVL 4.4–5.2% vs. 0.058–0.080); several large dark brown patches along both ventrolateral sides of abdomen (vs. dark brown broad stripe); inner metacarpal tubercle, outer metacarpal tubercle, inner metatarsal tubercle, and tip of fingers and toes ivory (vs. orange).

In having a moderate body size (male SVL 34.4–44.7 mm, female SVL 36.2–52.8 mm), *Boulenophrys gutu* **sp. nov.** differs from smaller species *B. acuta*, *B. angka*, *B. baishanzuensis*, *B. cheni*, *B. congjiangensis*, *B. daiyunensis*, *B. daoji*, *B. elongata*, *B. frigida*, *B. gaolanensis*, *B. hungtai*, *B. jiulianensis*, *B. kuatunensis*, *B. meihuamontis*, *B. rubrimera*, *B. sanmingensis*, *B. shimentaina*, *B. shunhuangensis*, *B. tongboensis*, and *B. wuliangshanensis*; and the new species differ from larger species *B. binlingensis*, *B. caudoprocta*, *B. fanjingmontis*, *B. jingdongensis*, *B. liboensis*, *B. mirabilis*, *B. omeimontis*, *B. qianbeiensis*, *B. sangzhiensis*, *B. shuichengensis*, and *B. spinata*.

In having weak vomerine ridge, Boulenophrys gutu **sp. nov.** differs from following species with without vomerine ridge: *B. baolongensis*, *B. binchuanensis*, *B. boettgeri*, *B. chishuiensis*, *B. dupanglingensis*, *B. lichun*, *B. lishuiensis*, *B. leishanensis*, *B. minor*, *B. ombrophila*, *B. tuberogranulatus*, *B. wushanensis*, *B. xuefengmontis*.

In absence of vomerine teeth, Boulenophrys gutu **sp. nov.** differs from species with vomerine teeth: *B. brachykolos*, *B. daweimontis*, *B. dongguanensis*, *B. fansipanensis*, *B. fengshunensis*, *B. hoanglienensis*, *B. insularis*, *B. jinggangensis*, *B. nankunensis*, *B. nanlingensis*, *B. palpebralespinosa*, *B. puningensis*, *B. yingdeensis*.

In heels meeting when thighs are positioned at right angles to body, *Boulenophrys gutu* **sp. nov.** differs from species with heels not meeting: *B. obesa*, *B. pepe*, and *B. wugongensis*; and differs from species with heels overlapping *B. anlongensis*, *B. changyangensis*, *B. daxuemontis*, *B. mufumontana*, *B. lushuiensis*, *B. xianjuensis*, *B. yangmingensis*, *B. yezhongensis*.

In having narrow lateral fringes on toes, *Boulenophrys gutu* **sp. nov.** differs from species without lateral fringes on toes: *B. caobangensis*, *B. jiangi*, *B. yaoshanensis*; and species with wide lateral fringes on toes: *B. yunkaiensis*, *B. lini*, *B. xiangnanensis*.

Detailed comparisons for several selected characters were listed Table S4.

**Figure 8:**
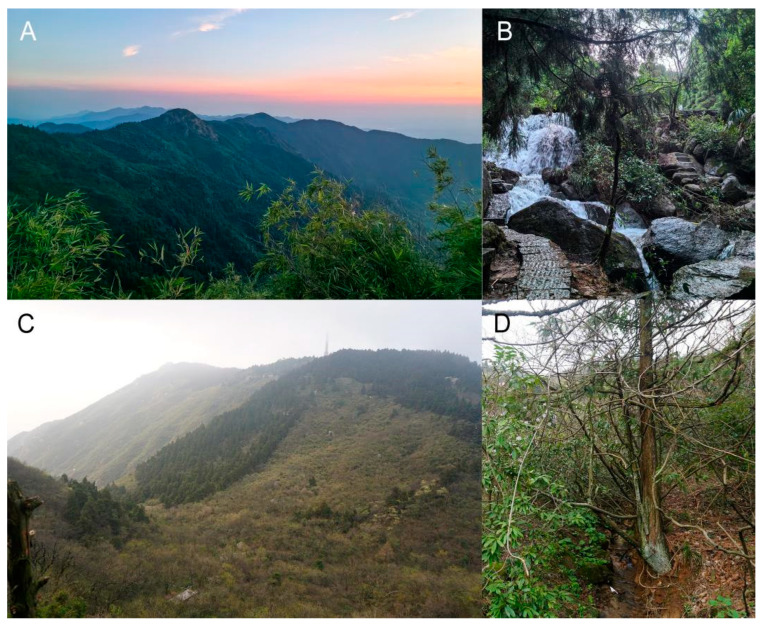
Habitats of *Boulenophrys hengshanensis* and *B. gutu* **sp. nov.** (**A**) landscape and forest of Mt. Hengshan; (**B**) the mountain stream at the site where the holotype of *B. gutu* **sp. nov.** was collected; (**C**) a moist bush valley near the top of Mt. Hengshan, where *B. hengshanensis* was found; (**D**) A small stream at Laotaiyi, where the female paratype of *B. hengshanensis* HUNL 1997:6–17 was collected.

**Ecological notes.** The new species inhabits the montane forest of Mt. Hengshan, found near streams at elevations ca. 250 m to ca. 1100 m (Figure 8). Advertisement calls were heard from April to July. *Quasipaa boulengeri* (Günther, 1889), *Amolops sinensis* Lyu, Wang and Wang, 2019, and *Boulenophrys hengshanensis* were found to be sympatric.

## 4. Discussion

The importance of examination of type specimens in taxonomic research is once again highlighted in the case of *Boulenophrys gutu* **sp. nov.** and *B. hengshanensis*. In morphologically conservative groups like *Boulenophrys*, it is necessary to make a taxonomic decision based on both morphological and molecular evidence.

The number of *Boulenophrys* species in Hunan Province, China, has now reached 19: *B. caudoprocta*, *B. cheni*, *B. dupanglingensis*, *B. gutu sp. nov.*, *B. hengshanensis*, *B. jinggangensis*, *B. lini*, *B. mufumontana*, *B. nanlingensis*, *B. ombrophila*, *B. sangzhiensis*, *B. shimentaina*, *B. shunhuangensis*, *B. tuberogranulata*, *B. wugongensis*, *B. xiangnanensis*, *B. xuefengmontis*, *B. yangmingensis* and *B. jiulianensis* (Figure 1) [2,11,13]. Most of these species were described recently. This indicates further research on the diversity of this group in Hunan Province could reveal more new findings.

The distribution of *Boulenophrys gutu* **sp. nov.** and *B. hengshanensis* overlap at elevation from ca. 700 m to ca. 1100 m according to multiple field investigations from 2018 to 2025, which have covered all four seasons. Although they are partially sympatric, their breeding seasons do not overlap. Calls of *B. hengshanensis* were heard from late September to early April, while calls of *B. gutu* **sp. nov.** were heard from early May to late July. This is suggested to be one of the factors for their prezygotic isolation.

Biodiversity could not be well protected without an accurate taxonomic framework. *Boulenophrys hengshanensis* is actually an endemic species only found above ac. 700 m on Mt. Hengshan. The overlooking of *B. gutu* **sp. nov.** will hinder its conservation policy decision. Further research on life history, distribution, and population size are recommended to support the conservation of these endemic species.

## 5. Conclusions

The type specimens of the recently described species *Boulenophrys hengshanensis* actually included specimens of two species. The specimens collected from Yanshou village in Mt. Hengshan in 2021 should be allocated to the new species, *B. gutu* **sp. nov.**

The description of *Boulenophrys hengshanensis* is revised based on the holotype (HUNL 0706000A), the female paratype (HUNL 1997:6–17) and nine newly collected specimens provided in this study.

## Figures and Tables

**Table 1:** Morphometrics of examined specimens of *Boulenophrys* from Mt. Hengshan in this research (*p*-Values < 0.05 were present in bold).

Measurements	*B. gutu* **sp. nov**				*B. hengshanensis*			*p*-Values from ANOVA in Males *
	Males (*n* = 7)		Females (*n* = 8)		Males (*n* = 10)		Females (*n* = 1)	
	Range	Mean ± SD	Range	Mean ± SD	Range	Mean ± SD		
SVL	34.4–44.7	38.8 ± 3.5	36.2–52.8	45 ± 7.1	34.4–39.3	37.4 ± 1.2	48.4	0.24
HL	10.3–13.7	12.2 ± 1.1	12–15.5	13.7 ± 1.3	10.6–13.5	12.2 ± 0.8	12.6	0.13
HW	11.7–15.9	14.3 ± 1.3	13.4–17.9	15.5 ± 1.9	12.7–15.2	14 ± 0.7	16.5	0.44
SL	4.1–5.5	4.7 ± 0.5	4.4–5.9	5.2 ± 0.6	3.8–4.8	4.3 ± 0.3	5	0.14
SND	2.1–2.6	2.4 ± 0.2	2.2–3.1	2.7 ± 0.3	1.6–2.6	2.2 ± 0.3	2	0.19
END	2.1–3.0	2.4 ± 0.3	2.1–3.1	2.6 ± 0.4	1.7–2.4	2.1 ± 0.2	2.3	**0.04**
IND	3.4–4.6	4.2 ± 0.4	3.7–5.2	4.4 ± 0.6	3.4–4.9	4 ± 0.4	5.5	0.53
IOD	3.5–4.5	4.1 ± 0.3	3.5–5.4	4.3 ± 0.7	3.8–4.6	4.2 ± 0.3	4.8	0.10
EL	4.3–5.4	5.1 ± 0.4	4.4–6.2	5.2 ± 0.7	3.8–4.9	4.3 ± 0.3	6.2	**0.00**
UEW	2.9–4.2	3.6 ± 0.4	3.3–5.1	4.2 ± 0.7	2.6–3.7	3.1 ± 0.4	3.7	0.08
IFE	6–8.3	7.3 ± 0.8	4.3–9.2	7.7 ± 1.8	5.9–7.4	6.5 ± 0.4	8.1	**0.02**
IBE	9.1–12.1	11.1 ± 1	10–14	12.1 ± 1.7	9.5–12.1	10.9 ± 0.8	13.8	0.57
TYD	1.8–3	2.7 ± 0.4	2.1–4.2	2.9 ± 0.8	1.9–2.9	2.3 ± 0.3	3.3	0.05
TYE	1.4–2.8	1.9 ± 0.4	1.7–2.9	2.2 ± 0.5	2.2–3.2	2.7 ± 0.4	3.6	**0.00**
FAL	6.3–9.7	8.3 ± 1	7.7–9.8	8.8 ± 0.9	7.5–8.7	8.1 ± 0.4	10.1	0.66
HAL	8.3–10.4	9.5 ± 0.9	8.9–12.2	10.6 ± 1.3	8.5–10.3	9.4 ± 0.6	10	0.27
FIL	2–2.9	2.6 ± 0.3	2.5–3.6	3.1 ± 0.4	2–2.9	2.6 ± 0.3	2.3	0.82
FIIL	2.2–2.9	2.6 ± 0.3	2.6–3.5	3.1 ± 0.3	2.3–2.9	2.6 ± 0.2	2.4	0.96
FIIIL	4–5.4	4.7 ± 0.5	4.3–5.7	5.1 ± 0.5	3.8–4.9	4.4 ± 0.4	5	0.52
FIVL	2.7–3.5	3.1 ± 0.3	2.9–4.2	3.7 ± 0.4	2.7–3.9	3.1 ± 0.3	3.4	0.55
FIIIW	0.6–1	0.9 ± 0.1	0.6–1	0.8 ± 0.1	0.6–1.1	0.8 ± 0.2	1	0.62
HLL	47.4–61.5	54.8 ± 4.8	51.7–68.6	60.5 ± 6.4	46–55.5	51 ± 3.4	54.6	0.13
FML	14.8–18.8	17.3 ± 1.5	16–21.7	19.2 ± 2.4	14.2–18.1	16.5 ± 1.3	19.7	0.65
TIL	15.3–19.4	17.1 ± 1.4	16.3–20.7	18.6 ± 1.8	14.5–16.6	15.6 ± 0.7	18.1	**0.00**
TFOL	19.8–27	23.7 ± 2.7	22.2–30.4	26.2 ± 3	20.6–24.2	22.6 ± 1.2	26.2	0.63
FOL	12.6–16.6	14.8 ± 1.6	14.2–19	16.9 ± 1.7	13.4–16.5	15 ± 1.1	18.7	0.06
IMT	1.5–2.2	1.8 ± 0.2	1.7–2.6	2.1 ± 0.3	2–3.1	2.5 ± 0.4	3.6	**0.00**
TYD/EL	0.42–0.58	0.53 ± 0.05	0.47–0.79	0.56 ± 0.11	0.42–0.61	0.54 ± 0.05	0.53	\
TIL/SVL	0.43–0.46	0.44 ± 0.01	0.37–0.45	0.42 ± 0.03	0.40–0.44	0.42 ± 0.01	0.37	\
FOL/SVL	0.36–0.40	0.38 ± 0.02	0.35–0.43	0.38 ± 0.03	0.37–0.43	0.40 ± 0.02	0.39	\
TFOL/SVL	0.57–0.67	0.61 ± 0.03	0.54–0.66	0.59 ± 0.04	0.57–0.64	0.60 ± 0.02	0.54	\
IMT/SVL	0.04–0.05	0.05 ± 0.005	0.04–0.06	0.05 ± 0.006	0.06–0.08	0.07 ± 0.008	0.07	\

* Based on the ratio of measurements of each characters to SVL.

## Data Availability

The original contributions presented in the study are included in the article/Supplementary Materials, further inquiries can be directed to corresponding author.

## Supplementary Materials

The following supporting information can be downloaded at: https://www.mdpi.com/article/10.3390/ani15182745/s1, Table S1: Samples used in this study. Table S2: Morphological comparisons of species of *Boulenophrys* [28,29,30,31,32,33,34,35,36,37,38,39,40,41,42,43,44,45,46,47,48,49,50,51,52,53,54,55,56,57,58,59,60,61,62,63]. Table S3: Genetic distance for Boulenophrys based on partial 16S rRNA gene sequences. Table S4: Genetic distance for *Boulenophrys* based on COI gene sequences. Table S5: Morphological measurements of *Boulenophrys gutu* sp. nov and *B. hengshanensis* examined in this study.
